# Characterising the interaction between the COPII component SEC24C and the human serotonin transporter

**DOI:** 10.1186/1471-2210-11-S2-A31

**Published:** 2011-09-05

**Authors:** Florian Koban, Sonja Sucic

**Affiliations:** 1 Institute of Pharmacology, Center of Physiology and Pharmacology, Medical University of Vienna, 1090 Vienna, Austria

## Background

The serotonin transporter (SERT) belongs to the SLC6 family of neurotransmitter transporters, which mediate reuptake of previously released neurotransmitters from the synapse. Mutation of C-terminus residues RI607–608 to alanine results in intracellular retention of SERT [1]. We subsequently showed that SERT depends on the COPII component SEC24C for its ER export and proposed RI607–608 as a putative interaction site on SERT for SEC24 proteins [2]. The aim of our current study is to characterise the nature of ER export of monoamine transporters.

## Methods

Using siRNAs to knock down SEC24 isoforms A–D in HeLa cells, we screened a series of double and truncation mutants generated along the C-terminus of SERT. HeLa cells were transfected with Sec24 siRNAs and, after 48 h, with YFP-tagged transporter plasmids. Functional effects of SEC24A–D knockdowns were determined by substrate uptake assays.

## Results

Export of the IK(609,610)AA-SERT mutant was not sensitive to knockdown of Sec24C. Remarkably, the closely related transporters for dopamine (DAT) and noradrenaline (NET), rely on Sec24D, and not C, for their ER export [2]. Accordingly, we replaced K610 by a tyrosine residue (Y) to switch the SERT export motif to a NET/DAT motif. The resulting K610Y-SERT mutant was more sensitive to the knockdown of SEC24D than of SEC24C. These observations predicted that SLC6 family members with a K-residue at the pertinent position ought to be clients of Sec24C. This prediction was verified by examining mGAT4.

## Conclusions

The data imply that residue K610 and the equivalent residues in other transporters specify which SEC24 paralogue is recruited for ER export. These export signals work independently because a concatemer of SERT and GAT-1 is affected by depletion of both SEC24C and SEC24D.
